# Propyl Gallate Attenuates Cognitive Deficits Induced by Chronic Sleep Deprivation Through Nrf2 Activation and NF-κB Inhibition

**DOI:** 10.3390/antiox15010079

**Published:** 2026-01-07

**Authors:** Xiangfei Zhang, Jingwen Cui, Liya Liu, Jing Sun, Bei Fan, Fengzhong Wang, Cong Lu

**Affiliations:** 1 Institute of Food Science and Technology, Chinese Academy of Agricultural Sciences, Beijing 100193, China; 82101235017@caas.cn (X.Z.); cuijingwen201207@163.com (J.C.); liuliya@caas.cn (L.L.); sunjing01@caas.cn (J.S.); fanbei@caas.cn (B.F.); 2 Institute of Food and Nutrition Development, Ministry of Agriculture and Rural Affairs, Beijing 100081, China

**Keywords:** propyl gallate, chronic sleep deprivation, cognitive deficits, antioxidant defense system, inflammatory cytokine response, Nrf2-driven antioxidant signaling

## Abstract

Chronic sleep deprivation (CSD) disrupts redox homeostasis and enhances neuroinflammatory activation, contributing to progressive cognitive impairment. Propyl gallate (PG), a lipophilic ester of gallic acid with established antioxidant activity, has not been investigated in the context of prolonged sleep deprivation. The current study examined whether PG alleviates CSD-induced oxidative imbalance, inflammatory activation, and associated behavioral deficits. Male ICR mice were subjected to 14 days of CSD using a rolling-drum apparatus and received oral PG (50, 100, or 200 mg/kg) or Ginkgo biloba extract (GBE, 40 mg/kg). Behavioral outcomes were assessed through a battery of tests, including the open-field, novel-object recognition, step-through, and Morris water maze paradigms. Oxidative and inflammatory biomarkers were assessed in serum and hippocampus, and Western blotting quantified the expression of nuclear factor erythroid 2–related factor 2 (Nrf2), heme oxygenase-1 (HO-1), NAD(P)H quinone oxidoreductase 1 (NQO1), nuclear factor-κB (NF-κB), inducible nitric oxide synthase (iNOS), and cyclooxygenase-2 (COX2). PG improved CSD-induced impairments in exploration, recognition memory, and spatial learning; restored antioxidant capacity; reduced lipid peroxidation; enhanced Nrf2-associated antioxidant signaling; and suppressed NF-κB-mediated inflammatory activation. These findings indicate that PG alleviates cognitive deficits induced by CSD through the modulation of redox homeostasis and neuroinflammatory responses, supporting its potential as an antioxidant derivative under chronic sleep-deprivation conditions.

## 1. Introduction

Sleep is essential for keeping neural systems balanced and for sustaining normal cognition [1,2]. However, with the increasing prevalence of stress, irregular schedules, and nighttime digital exposure, chronic insufficient sleep has become a widespread problem in modern societies [3,4]. Epidemiological evidence indicates that nearly one-third of adults experience insufficient or poor-quality sleep, which is closely associated with cognitive decline, emotional instability, and a heightened risk of neurodegenerative and psychiatric disorders [5]. The chronic sleep deprivation (CSD) model reliably reproduces hippocampal dysfunction and memory deficits observed in prolonged sleep loss, providing a well-established experimental paradigm for investigating the neural and molecular basis of sleep-related cognitive impairment [6,7].

Accumulating evidence shows that oxidative stress and neuroinflammation are the key contributors to neuropathological consequences of CSD [8,9]. Excessive production of reactive oxygen species (ROS) damages lipids, proteins, and nucleic acids, thereby impairing mitochondrial function and neuronal integrity [10,11]. Concurrently, activation of microglia stimulates the release of pro-inflammatory cytokines such as interleukin-1β (IL-1β), interleukin-6 (IL-6), and tumor necrosis factor-α (TNF-α), which further exacerbate oxidative injury [12,13]. The interplay between oxidative stress and inflammation creates a vicious cycle that amplifies neuronal damage and ultimately disrupts hippocampal synaptic plasticity, leading to learning and memory deficits [14,15]. Despite extensive research has elucidated the detrimental effects of CSD, effective pharmacological or nutritional strategies capable of simultaneously mitigating oxidative stress and neuroinflammation remain limited.

Among intrinsic protective mechanisms, the nuclear factor erythroid 2-related factor 2 (Nrf2) pathway serves as a principal controller of cellular oxidative equilibrium [16]. Nrf2 induces expression of antioxidant and detoxification enzymes including heme oxygenase-1 (HO-1) and NAD(P)H quinone oxidoreductase-1 (NQO1) [17]. When oxidative stress kicks in, NF-κB becomes more active, spurring the production of pro-inflammatory molecules such as inducible nitric oxide synthase (iNOS) and cyclooxygenase-2 (COX2), which, in turn, intensifies neural damage [18,19]. Therefore, the interplay between Nrf2-driven antioxidant responses and NF-κB-mediated inflammatory signaling critically determines neuronal resilience to oxidative challenges [20]. Compounds capable of simultaneously activating Nrf2 and suppressing NF-κB are therefore particularly promising for restoring redox–immune equilibrium in the sleep-deprived brain.

Propyl gallate (PG; propyl 3,4,5-trihydroxybenzoate), is an ester derivative of the natural polyphenol gallic acid. Esterification enhances the lipophilicity, chemical stability, and membrane permeability of the parent molecule, thereby improving bioavailability and potentially facilitating brain accessibility [21,22]. Previous studies have demonstrated that PG preserves mitochondrial integrity, inhibits lipid peroxidation, and alleviates oxidative injury in diverse cellular and tissue models [23,24]. In parallel, polyphenols have been widely reported to regulate redox homeostasis and modulate multiple signaling pathways involved in inflammatory responses [25,26]. However, whether PG exerts similar regulatory effects on oxidative and inflammatory signaling under CSD conditions, and how such modulation relates to cognitive impairment, remains insufficiently characterized.

Therefore, a CSD rodent model was used to assess the cognitive impact and molecular mechanisms of PG. Through the integration of behavioral appraisals alongside biochemical and molecular analyses, this investigation sheds new light on how PG modulates oxidative and inflammatory pathways during CSD conditions, establishing a solid scientific foundation for its potential use as a neuroprotective polyphenol in promoting cognitive well-being and developing functional food products.

## 2. Materials and Methods

### 2.1. Reagents and Biological Materials

Propyl gallate (PG, ≥98% purity) was obtained from Shanghai Yuanye Bio-Technology Co., Ltd. (Shanghai, China). Ginkgo biloba extract (GBE, approval no. HJ20140768) was provided by Dr. Willmar Schwabe GmbH & Co. KG (Karlsruhe, Germany); each tablet contained 40 mg extract, standardized to 9.6 mg flavonoid glycosides and 2.4 mg terpene lactones. Assay kits for T-AOC, SOD, lipid peroxidation, TNF-α, IL-6, and IL-1β were from Nanjing Jiancheng Bioengineering Institute (Nanjing, China). Primary antibodies were from Proteintech (Chicago, IL, USA), and secondary antibodies from Abcam (Cambridge, UK).

### 2.2. Ethical Statement

Male ICR mice (18–22 g, SPF; n = 60) were provided by Hunan Prima Pharmaceutical Research Center Co., Ltd. (Changsha, China). All animal procedures were approved by the Institutional Animal Care and Use Committee (IACUC) at Hunan Prima Pharmaceutical Research Center Co., Ltd. (approval number: HNSE2025(5)009; 10 February 2025). Animal care and experimental operations followed national guidelines for laboratory animal management.

Only male mice were used in this study. Female rodents exhibit cyclical fluctuations in ovarian hormones across the estrous cycle, which are known to influence sleep–wake regulation [27], hippocampal plasticity, oxidative stress responses, and neuroinflammatory signaling [28]. Adequately controlling for these hormone-dependent effects requires estrous cycle staging or hormonal manipulation, which introduces additional experimental complexity and variability. As the present work aimed to establish the mechanistic basis of PG-mediated neuroprotection under CSD conditions, a single-sex experimental design was employed to reduce biological heterogeneity and improve statistical power. The potential influence of sex differences was not addressed in this study and will be examined in future experiments with appropriate estrous cycle control.

### 2.3. Treatment Administration and Group Allocation

Mice were kept in an environment with regulated temperature (20–24 °C) and humidity (55 ± 10%), alternating light and dark phases for 12 h, and were provided with unrestricted access to nourishment and hydration. Six animals were housed per cage. After a 3-day acclimatization period, mice were weighed and randomly assigned to experimental groups using a random number table, ensuring comparable baseline body weights across groups.

The mice were allocated into six experimental groups (n = 10 per group):

(1) CON (Control): No stress treatment; animals received pure water by oral gavage each day;
(2) CSD (Model): Mice were subjected to the CSD protocol and given daily gavage of pure water;
(3) GBE (Positive Control): CSD-treated mice were administered GBE at 40 mg/kg/day;
(4) PG 50 mg/kg: CSD-treated mice were administered PG at 50 mg/kg/day;
(5) PG 100 mg/kg: CSD-treated mice were administered PG at 100 mg/kg/day;
(6) PG 200 mg/kg: CSD-treated mice were administered PG at 200 mg/kg/day.

The PG doses (50–200 mg/kg/day) were chosen based on prior in vivo studies reporting antioxidant and neuroprotective efficacy at 100–750 mg/kg in rodent models of ischemic or oxidative injury [29,30]. Considering the chronic nature of sleep deprivation, continuous dosing was applied. Toxicological evidence further shows that PG is well tolerated, with a 90-day no-observed-adverse-effect level (NOAEL) of 135 mg/kg/day in rats [31], confirming that the present doses are within a safe and pharmacologically relevant range.

GBE was used as a positive reference owing to its well-documented clinical benefits in alleviating mild cognitive impairment and supporting cerebrovascular health [32]. Preclinical studies have shown that a daily dose of 40 mg/kg of GBE can improve learning performance and memory retention, and has been reported to stimulate hippocampal neurogenesis in mice, thereby justifying its selection as the comparative treatment [33].

### 2.4. CSD Mouse Model Establishment

CSD was produced with an automated sleep-interruption system (KSYY-I18S418M1), which consists of multiple stainless-steel rotating drums controlled by a programmable interface. The device allows precise adjustment of rotation speed, pause intervals, and operation duration, enabling stable and reproducible disruption of sleep while minimizing excessive physical stress. Each chamber contained food and water to ensure ad libitum access during the procedure.

After a 3-day acclimation period, mice assigned to the CSD groups were individually into the rotating chambers. The cylinders operated at 1 revolution per minute, followed by a 2 min pause, with the direction of rotation changed randomly to prevent behavioral adaptation. Throughout the experiment, environmental parameters—including temperature (23 ± 2 °C), humidity (55 ± 10%), and a 12 h light/dark cycle—were kept constant.

**Figure 1:**
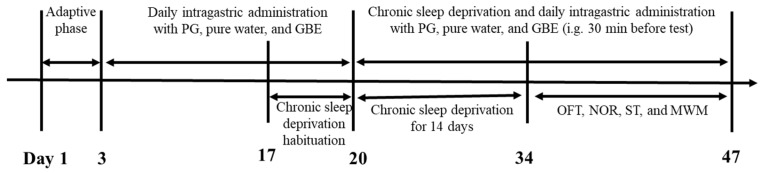
Experimental schedule for the CSD mouse model.

To minimize acute stress responses, animals underwent a 3 h daily adaptation session (Days 18–20), during which the apparatus operated with the same parameters used for formal modeling. Following adaptation, mice were subjected to 14 consecutive days of continuous sleep disruption (Days 21–34) under identical settings. Control mice were housed in identical cages placed outside the apparatus without motion interference. An overview of the experimental timeline is presented in Figure 1.

### 2.5. Behavioral Tests

#### 2.5.1. Open Field Test (OFT)

On day 35 of the experimental schedule, spontaneous locomotor activity and exploratory behavior were assessed using the OFT, as previously described [34]. Mice were individually placed in the center of a square open-field arena (40 cm × 40 cm × 40 cm) and allowed to explore freely for 5 min under standard testing conditions. Behavioral data were collected using an automated animal behavior analysis system (DigBehv; Shanghai Jiliang Software Technology Co., Ltd., Shanghai, China). Average movement speed, total movement distance, and the center-to-periphery time ratio were recorded and used for behavioral analysis.

#### 2.5.2. Novel Object Recognition Test (NOR)

On days 36–38, recognition memory was evaluated using the NOR test, as previously described [35]. The test consisted of habituation, familiarization, and test phases conducted over three consecutive days according to established protocols. Briefly, mice were exposed to two identical objects during the familiarization phase and, following a retention interval, one familiar object was replaced with a novel object during the test phase. Behavioral data were recorded and analyzed using a novel object recognition video analysis system (model: JL Behv-ORG-4; Shanghai Jiliang Software Technology Co., Ltd., Shanghai, China). Exploration time spent with the familiar object (T_F_) and the novel object (T_N_) was automatically quantified. Recognition performance was expressed as the discrimination index (DI), calculated as DI = (T_N_ − T_F_)/(T_N_ + T_F_).

#### 2.5.3. Step-Through Test (ST)

On days 40–41, learning and memory performance were assessed using the ST passive avoidance test, as previously described [36]. The test was conducted in an apparatus consisting of an illuminated compartment and a dark compartment separated by a guillotine door. During the training phase, mice were placed in the illuminated compartment, and upon entering the dark compartment, a mild foot shock was delivered. Twenty-four hours later, memory retention was evaluated in the test phase without shock exposure. Behavioral data were collected using the same automated animal behavior analysis system as described for the OFT (DigBehv; Shanghai Jiliang Software Technology Co., Ltd., Shanghai, China). Latency to enter the dark compartment and the number of errors (re-entries into the dark compartment) were recorded and used as indices of learning and memory performance.

#### 2.5.4. Morris Water Maze Test (MWM)

Spatial learning and memory were assessed using the MWM test between days 42 and 47, as previously described [37,38]. The test consisted of an acquisition (training) phase followed by a probe trial. During the training phase, mice were trained to locate a hidden platform submerged beneath the water surface over five consecutive days. In the probe trial, the platform was removed, and mice were allowed to swim freely to evaluate memory retention. Behavioral data were collected using an automated Morris water maze video-tracking system (model: WMT-100S; Shanghai Jiliang Software Technology Co., Ltd., Shanghai, China). Escape latency and swimming distance were recorded during the training phase, while time spent in the target quadrant, number of platform crossings, and average swimming speed were measured during the probe trial to assess spatial learning and memory performance.

### 2.6. Samples Collection

Following the conclusion of the behavioral experiments, the rodents were subjected to an overnight fasting period before being anesthetized using a 10% solution of chloral hydrate. Blood was drawn from the retro-orbital sinus, allowed to clot at room temperature, and then centrifuged at 3000 rpm for 15 min at 4 °C to obtain serum. The separated serum was stored at −80 °C until biochemical measurements were conducted.

Immediately after blood collection, the brain was removed on ice. The hippocampus from each mouse was dissected and divided into two fractions. One portion was homogenized in pre-chilled PBS (pH 7.4) for assays of oxidative stress and inflammatory markers, while the other was snap-frozen in liquid nitrogen for later Western blot analysis.

### 2.7. Biochemical and Immunoassays

Oxidative stress–related parameters in serum and hippocampal homogenates were determined using standardized biochemical assays. T-AOC was measured based on the ferric reducing antioxidant power principle, reflecting the overall antioxidant defense capacity of the samples. SOD activity was determined using the xanthine oxidase method, which evaluates the enzyme’s ability to scavenge superoxide anions. Lipid peroxidation was assessed by measuring thiobarbituric acid reactive substances (TBARS).

Pro-inflammatory cytokines, including IL-1β, IL-6, and TNF-α, were quantified using enzyme-linked immunosorbent assay (ELISA). Absorbance values were measured with a microplate reader, and cytokine concentrations were calculated from standard curves generated for each assay.

All biochemical and immunoassays were performed in accordance with the manufacturers’ instructions, and assay results were normalized as required for subsequent statistical analysis.

### 2.8. Analysis by Western Blotting

Hippocampal tissue was processed using a chilled RIPA lysis buffer with protease and phosphatase inhibitor mixtures. The homogenates were clarified by high-speed centrifugation, and the resulting supernatants were subjected to protein determination using a BCA assay. Extracted protein was mixed with loading buffer, denatured through 5 min heating, and separated via 10–12% SDS–polyacrylamide gels. Proteins were transferred onto PVDF membranes via electrotransformation post-electrophoresis.

Antibodies targeting the proteins were added to the TBST-blocked membranes, which were incubated at 4 °C for 16 h after a 1 h room temperature exposure to 5% non-fat milk. Membranes, post-washing, were incubated with suitable HRP-linked secondary antibodies for an hour and then detected via enhanced chemiluminescence. Captured with an imaging system, band intensities were subsequently processed via Image-Pro Plus 6.0.

### 2.9. Data Analysis

Statistical analyses were performed using SPSS 22.0 (IBM, Armonk, NY, USA) and GraphPad Prism 8.0.2 (GraphPad Software, San Diego, CA, USA). Data are presented as mean ± SD. Prior to statistical comparisons, data distribution was assessed using the Shapiro–Wilk test for normality, and homogeneity of variances was evaluated using Levene’s test. As the data met the assumptions of normality and homoscedasticity, group differences were analyzed using one-way analysis of variance (ANOVA), followed by Fisher’s least significant difference (LSD) test for post hoc comparisons. A *p*-value < 0.05 was considered statistically significant. The detailed quantitative data supporting these results are provided in Tables S1–S28.

## 3. Results

### 3.1. Effects of PG Intervention on Locomotor and Exploratory Behaviors in CSD Mice

**Figure 2:**
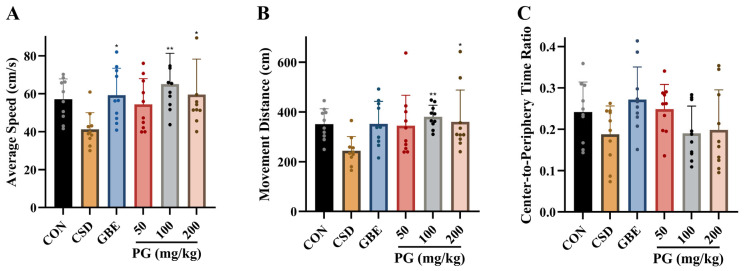
PG ameliorates locomotor and exploratory deficits induced by CSD. (**A**) Average movement speed in the OFT is shown, representing the animals’ basic locomotor capacity. (**B**) Total distance traveled is presented as an additional measure of spontaneous activity levels. (**C**) The center-to-periphery time ratio is displayed, an index commonly used to evaluate exploratory behavior and anxiety-related responses. Data are mean ± SD (*n* = 10). * *p* < 0.05, ** *p* < 0.01 vs. CSD.

As shown in Figure 2, CSD decreased average movement speed and total movement distance in the open field, although these reductions did not reach statistical significance. GBE (40 mg/kg) significantly increased locomotor speed (*p* < 0.05). PG treatment also enhanced locomotor activity, with significant increases in average speed observed at 50, 100, and 200 mg/kg (*p* < 0.05–0.01), and modest increases in total movement distance at all doses (*p* < 0.05–0.01). The central-to-peripheral time ratio decreased for the CSD group, with no notable variations between groups. Overall, PG improved several indices of locomotor activity suppressed by CSD, while effects on center exploration were not statistically significant.

### 3.2. Effects of PG on Object Recognition in CSD Mice

**Figure 3:**
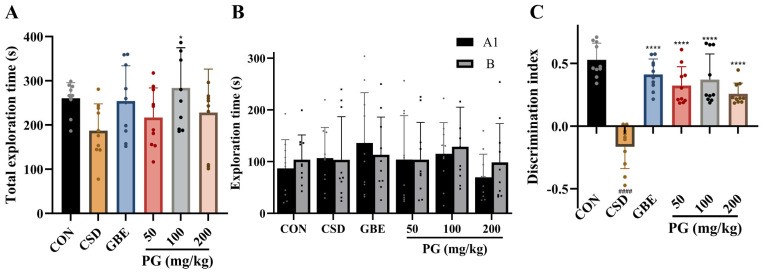
PG improves recognition memory deficits associated with CSD. (**A**) Total exploration time in the NOR task is presented to indicate general exploratory engagement during the test session. (**B**) Exploration duration directed toward the familiar object (A1) and the novel object (B) is shown, reflecting the animals’ preference for novelty. (**C**) The discrimination index, calculated from the relative exploration of the two objects, is displayed as a quantitative measure of recognition memory performance. Data are mean ± SD (*n* = 10). ^####^ *p* < 0.0001 vs. CON; * *p* < 0.05, **** *p* < 0.0001 vs. CSD.

As shown in Figure 3, mice subjected to CSD exhibited a marked impairment in recognition memory, as evidenced by a significant reduction in the discrimination index compared with the control group (*p* < 0.0001). Although the total exploration time showed a decreasing trend in CSD mice, this change did not reach statistical significance. Treatment with GBE (40 mg/kg) significantly improved recognition performance, as reflected by a robust increase in the discrimination index (*p* < 0.0001). Similarly, PG administration at doses of 50, 100, and 200 mg/kg significantly reversed the CSD-induced decline in discrimination index (*p* < 0.0001), indicating a clear protective effect of PG on recognition memory. Among the PG-treated groups, the 100 mg/kg dose exhibited the most pronounced improvement, while the 50 mg/kg and 200 mg/kg groups also showed substantial and comparable enhancement. Analysis of object exploration patterns revealed a preferential exploration of the novel object (B) over the familiar object (A1) in PG- and GBE-treated mice, further supporting the conclusion that PG effectively ameliorates recognition memory deficits induced by CSD.

### 3.3. Effects of PG Intervention on Learning and Memory Performance in CSD Mice

**Figure 4:**
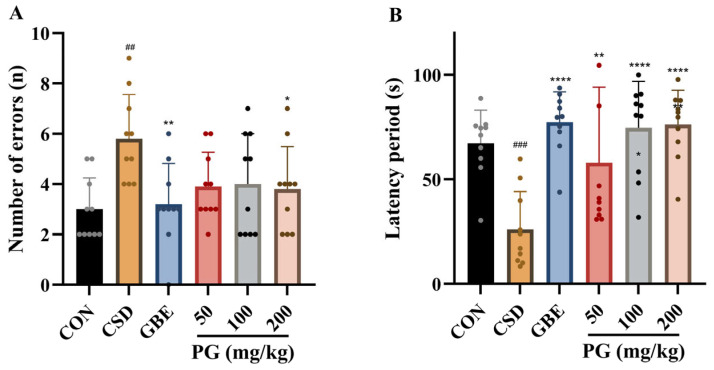
PG alleviates learning and memory impairments in the ST following CSD. (**A**) The number of errors made during the retention trial is shown, reflecting the animals’ ability to recall the aversive stimulus associated with the dark compartment. (**B**) Latency to enter the dark compartment is presented as a measure of memory retention, with longer latency indicating better recall of the learned avoidance response. Data are mean ± SD (*n* = 10). ^##^ *p* < 0.01, ^###^ *p* < 0.001 vs. CON; * *p* < 0.05, ** *p* < 0.01, **** *p* < 0.0001 vs. CSD.

The results depicted in Figure 4 reveal that CSD mice displayed notable deficiencies in the ST, characterized by a significant increase in the number of errors (*p* < 0.01) and a marked reduction in latency period compared with the control group (*p* < 0.001), indicating impaired passive avoidance memory. Treatment with GBE (40 mg/kg) significantly improved ST performance, as evidenced by a reduction in error frequency (*p* < 0.01) and a pronounced prolongation of latency period (*p* < 0.0001). PG administration produced dose-dependent but parameter-specific effects. All three doses of PG (50, 100, and 200 mg/kg) significantly increased latency periods (*p* < 0.01–0.0001), whereas only the 200 mg/kg dose significantly reduced the number of errors (*p* < 0.05). PG50 and PG100 did not produce statistically significant changes in error counts. Overall, PG partially ameliorated CSD-induced impairments in passive avoidance behavior, with latency period appearing more sensitive to PG treatment than error frequency, and the higher dose (200 mg/kg) exerting a broader behavioral improvement.

### 3.4. Effects of PG on Spatial Navigation and Memory in CSD Mice

As shown in Table 1 and Table 2, mice subjected to CSD displayed significantly impaired spatial learning performance, as evidenced by longer swimming distances and escape latencies compared with the control group (*p* < 0.01–0.0001). Treatment with GBE (40 mg/kg) markedly improved these parameters from day 3 onward (*p* < 0.01–0.001). Similarly, PG (50–200 mg/kg) produced dose-dependent enhancements across successive training days, with the 50 mg/kg and 100 mg/kg groups showing significant improvement from day 2 and the 200 mg/kg group maintaining reductions through day 5 (*p* < 0.001–0.0001). These findings indicate that PG effectively enhances spatial learning acquisition and navigation efficiency in CSD-induced cognitive impairment.

**Figure 5:**
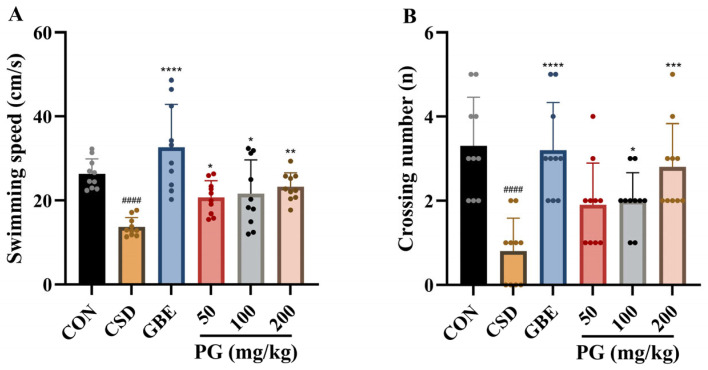
PG improves spatial search performance during the MWM probe test following CSD. (**A**) Swimming speed during the probe trial is shown, reflecting general locomotor capability and search efficiency in the maze. (**B**) The number of platform crossings is presented as an indicator of spatial memory retention, with higher values corresponding to better recall of the platform location. Data are mean ± SD (*n* = 10). ^####^ *p* < 0.0001 vs. CON; * *p* < 0.05, ** *p* < 0.01, *** *p* < 0.001, **** *p* < 0.0001 vs. CSD.

As shown in Table 3 and Figure 5, CSD markedly impaired spatial memory retention, as evidenced by reduced dwell time in the target quadrant (Q1), increased occupancy of the opposite quadrant (Q3), slower swimming speed, and fewer platform crossings compared with control mice (*p* < 0.001). Treatment with the positive control GBE (40 mg/kg) significantly improved all probe trial parameters, including increased Q1 dwell time, reduced Q3 occupancy, and enhanced swimming speed and platform crossings (*p* < 0.001). PG administration (50–200 mg/kg) partially reversed CSD-induced deficits. PG at 50 mg/kg produced the most pronounced improvement, significantly increasing Q1 dwell time and swimming speed and reducing Q3 occupancy (*p* < 0.01–0.001), whereas PG at 100 mg/kg and 200 mg/kg showed moderate but significant improvements in selected parameters (*p* < 0.05–0.001). Overall, PG ameliorated spatial memory impairments in the probe trial, with the 50 mg/kg dose showing the greatest efficacy.

### 3.5. Effects of PG on Serum and Hippocampal Oxidative Stress in CSD Mice

**Figure 6:**
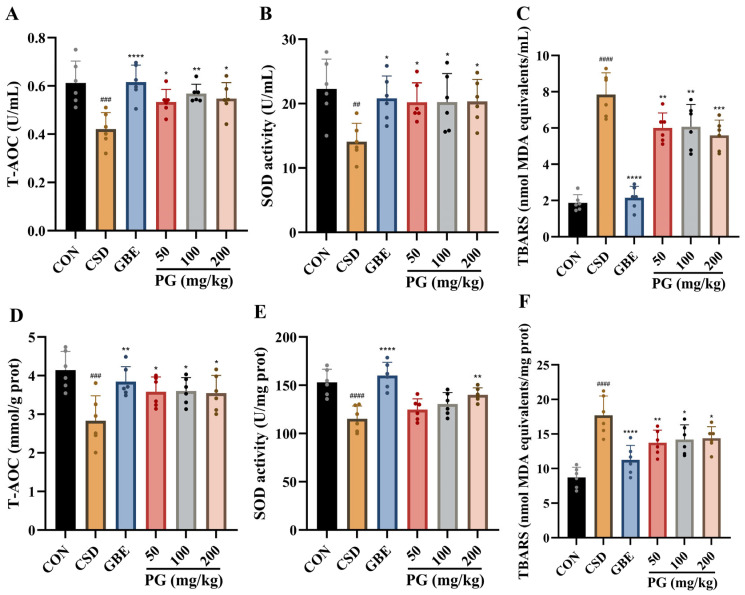
PG regulates oxidative stress status in both serum and hippocampal tissues of mice subjected to CSD. (**A**–**C**) Serum T-AOC, SOD activity, and lipid peroxidation levels, assessed by TBARS, reflecting systemic redox status. (**D**–**F**) Corresponding measurements in hippocampal tissues illustrate local oxidative stress alterations and the restorative effects of PG treatment under CSD conditions. Data are mean ± SD (*n* = 6). ^##^ *p* < 0.01, ^###^ *p* < 0.001, ^####^ *p* < 0.0001 vs. CON; * *p* < 0.05, ** *p* < 0.01, *** *p* < 0.001, **** *p* < 0.0001 vs. CSD.

As shown in Figure 6, CSD markedly disrupted redox homeostasis, as evidenced by significant reductions in T-AOC and SOD activity and marked increases in lipid peroxidation, reflected by elevated TBARS levels, in both serum and hippocampal tissues (*p* < 0.01–0.0001). Treatment with GBE (40 mg/kg) significantly reversed these oxidative alterations in both compartments (*p* < 0.05–0.001). PG administration attenuated CSD-induced oxidative stress. Across all tested doses (50–200 mg/kg), PG significantly enhanced antioxidant capacity, as indicated by increased T-AOC and SOD activity, and reduced TBARS levels in serum and hippocampus (*p* < 0.05–0.001). Overall, PG alleviated oxidative damage induced by CSD by strengthening antioxidant defenses and limiting lipid peroxidation.

### 3.6. Effects of PG on Nrf2/HO-1-Mediated Antioxidant Defense in the Hippocampus Under CSD

**Figure 7:**
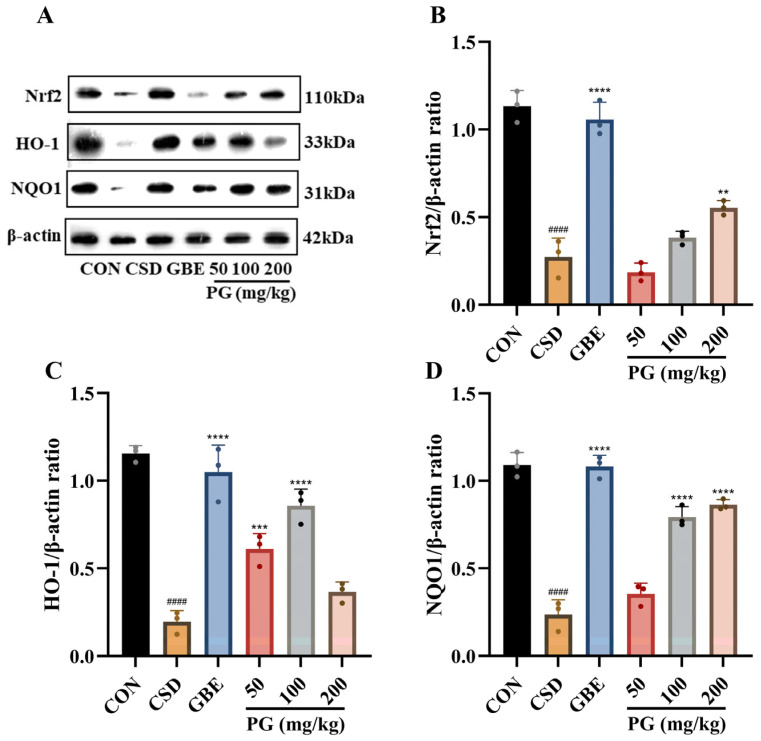
PG enhances Nrf2–HO-1–NQO1 antioxidant signaling in the hippocampus under CSD. (**A**) Representative Western blot bands and quantitative analyses of (**B**) Nrf2, (**C**) HO-1, and (**D**) NQO1 protein expression normalized to β-actin. Data are mean ± SD (*n* = 3). ^####^ *p* < 0.0001 vs. CON; ** *p* < 0.01, *** *p* < 0.001, **** *p* < 0.0001 vs. CSD.

As shown in Figure 7, CSD markedly reduced the hippocampal protein expression of Nrf2, HO-1, and NQO1 compared with the control group (*p* < 0.001–0.0001). Treatment with GBE (40 mg/kg) significantly restored the expression of all three proteins (*p* < 0.001–0.0001). PG treatment partially reversed CSD-induced suppression of the Nrf2 signaling pathway in a marker- and dose-dependent manner. Nrf2 protein expression was significantly increased only in the 200 mg/kg PG group (*p* < 0.05), whereas the 50 mg/kg and 100 mg/kg groups showed no significant changes. HO-1 expression was significantly elevated by PG at 50 mg/kg (*p* < 0.05) and more robustly at 100 mg/kg (*p* < 0.01), while no significant effect was observed at 200 mg/kg. For NQO1, significant increases were detected in the 100 mg/kg and 200 mg/kg PG groups (*p* < 0.01), whereas the 50 mg/kg group showed no significant difference from the CSD group. Overall, PG mitigated CSD-induced downregulation of Nrf2-related antioxidant proteins in the hippocampus, although the extent of recovery differed among individual targets and doses.

### 3.7. Effects of PG on Serum and Hippocampal Pro-Inflammatory Cytokine in CSD Mice

**Figure 8:**
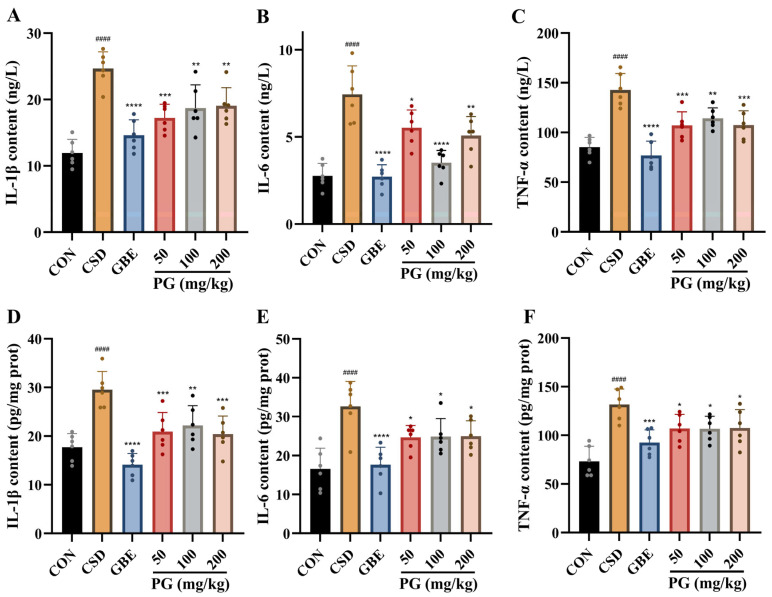
PG mitigates serum and hippocampal inflammatory cytokine elevations induced by CSD. (**A**–**C**) Serum and (**D**–**F**) hippocampal levels of IL-1β, IL-6, and TNF-α. Data are mean ± SD (*n* = 6). ^####^ *p* < 0.0001 vs. CON; * *p* < 0.05, ** *p* < 0.01, *** *p* < 0.001, **** *p* < 0.0001 vs. CSD.

Figure 8 shows that CSD induced a pronounced inflammatory response, as evidenced by significant increases in IL-1β, IL-6, and TNF-α levels in both serum and hippocampal tissues compared with the control group (*p* < 0.01–0.0001). Treatment with GBE (40 mg/kg) significantly reduced the levels of all three pro-inflammatory cytokines in both compartments (*p* < 0.05–0.001). PG administration attenuated CSD-induced inflammatory activation in both serum and hippocampus. In serum, PG at doses of 50–200 mg/kg significantly decreased IL-1β, IL-6, and TNF-α levels (*p* < 0.05–0.01). Similarly, in the hippocampus, PG treatment significantly reduced IL-1β, IL-6, and TNF-α across all tested doses (*p* < 0.05–0.01). Overall, PG effectively counteracted the CSD-induced elevation of pro-inflammatory cytokines in both peripheral circulation and brain tissue, indicating a broad anti-inflammatory effect under CSD conditions.

### 3.8. Effects of PG on NF-κB p65-Driven Inflammatory Signaling in the Hippocampus Under CSD

**Figure 9:**
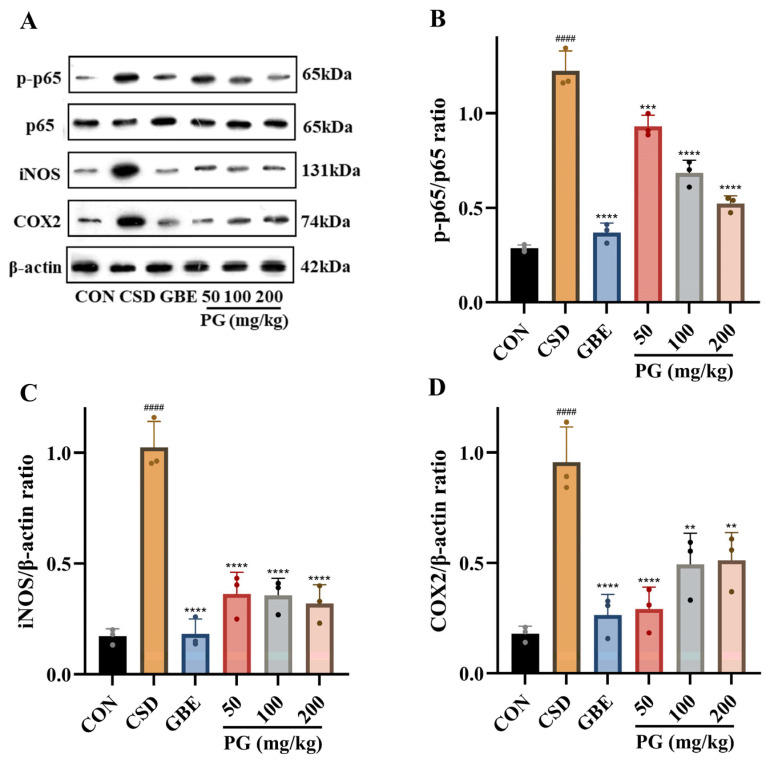
PG suppresses NF-κB–related inflammatory responses in the hippocampus of CSD mice. (**A**) Representative Western blot images showing p-p65, p65, iNOS, COX2, and β-actin protein bands. Quantitative analyses of (**B**) the p-p65/p65 ratio, (**C**) iNOS/β-actin ratio, and (**D**) COX2/β-actin ratio are presented. Data are expressed as mean ± SD (*n* = 3). ^####^ *p* < 0.0001 vs. CON; ** *p* < 0.01, *** *p* < 0.001, **** *p* < 0.0001 vs. CSD. The same β-actin bands are shown in Figure 7 and Figure 9 because the corresponding target proteins were analyzed using the same set of samples under identical experimental conditions.

As shown in Figure 9, CSD markedly increased NF-κB p65 phosphorylation and significantly elevated the hippocampal expression of iNOS and COX2 compared with the control group (*p* < 0.001–0.0001). Treatment with GBE (40 mg/kg) significantly suppressed NF-κB activation and reduced iNOS and COX2 expression (*p* < 0.001–0.0001). PG administration (50–200 mg/kg) effectively attenuated CSD-induced activation of the NF-κB signaling pathway, as evidenced by significant reductions in p-p65/p65 ratios and decreased expression of iNOS and COX2 (*p* < 0.01–0.0001). The inhibitory effects of PG were comparable among the tested doses, indicating a consistent anti-inflammatory response rather than a strict dose-dependent pattern. Collectively, PG mitigated hippocampal neuroinflammatory activation induced by CSD.

## 4. Discussion

In this study, we examined the effects of PG, a lipophilic ester of gallic acid, on CSD-induced behavioral and molecular disturbances. PG administration ameliorated cognitive impairments induced by CSD, shown by improvements in locomotor activity, recognition performance, passive-avoidance retention, and spatial learning, although the magnitude of behavioral improvement varied across tests and doses. PG also strengthened antioxidant defenses by restoring T-AOC and SOD activities and reducing lipid peroxidation, as indicated by decreased TBARS levels, accompanied by enhanced expression of Nrf2, HO-1, and NQO1. In parallel, PG attenuated neuroinflammatory responses, as reflected by decreased hippocampal levels of IL-1β, IL-6, and TNF-α and reduced expression of NF-κB–related inflammatory signaling proteins and enzymes, including phosphorylated p65 (p-p65), iNOS, and COX2. Collectively, these findings suggest that PG may mitigate CSD-induced cognitive deficits partly through modulation of oxidative and inflammatory pathways.

In modern societies, sleep deprivation often arises from lifestyle-associated factors such as shift work, excessive nighttime light exposure, stress, and anxiety [39]. Among experimental paradigms, CSD has been increasingly employed as a translational model to recapitulate such long-term sleep disturbances [40]. Compared with acute sleep restriction that primarily induces transient changes in arousal and attention, CSD imposes sustained oxidative and inflammatory stress that gradually compromises hippocampal synaptic plasticity, thereby disrupting neuronal signaling and impairing memory consolidation [41,42]. Consistent with the previous reports of cognitive and behavioral impairments following CSD [43,44], the CSD-exposed mice in this study exhibited reduced exploratory activity in the OFT, diminished recognition preference in the NOR task, and spatial learning deficits in the MWM. Administration of PG attenuated these behavioral abnormalities, as evidenced by improved locomotor activity, enhanced recognition preference, and shortened escape latency. However, these improvements were dose-dependent and task-specific, reflecting the heterogeneous impact of CSD on different learning and memory processes. The expected effects of GBE further confirm the validity of the CSD model and support the neuroprotective potential of PG.

Oxidative imbalance occurs due to an excess of reactive oxygen species and a deficit in antioxidant defenses [45], leading to molecular and cellular injury [46,47]. The activities of key antioxidant enzymes, such as SOD and T-AOC, indicate the capacity of endogenous redox defense, whereas MDA, generated from lipid peroxidation, serves as a reliable marker of oxidative injury [48,49]. These complementary indices are routinely employed to assess oxidative stress status in biological systems. In our study, CSD caused a clear collapse of antioxidant defenses, reflected by substantial reductions in T-AOC and SOD activity, together with a marked increase in lipid peroxidation, as indicated by elevated TBARS levels, demonstrating severe oxidative imbalance. These findings are consistent with previous reports showing that chronic sleep loss weakens endogenous antioxidant defenses and promotes oxidative stress [50,51]. PG supplementation restored SOD and T-AOC activity and attenuated lipid peroxidation, as evidenced by reduced TBARS levels, indicating an improvement in overall antioxidant status under CSD conditions. Notably, these biochemical improvements paralleled the behavioral benefits of PG, suggesting a functional relevance between redox restoration and cognitive performance.

At the molecular level, Nrf2, together with its downstream targets HO-1 and NQO1, represents a central defense module governing intracellular antioxidant balance [52]. When cells encounter oxidative challenge, Nrf2 is released from its inhibitory partner Keap1 and accumulates in the nucleus. There, it interacts with antioxidant response element (ARE) sequences to drive the expression of detoxifying and cytoprotective enzymes, including HO-1 and NQO1 [53,54]. In the CSD group, the protein expression levels of Nrf2, HO-1, and NQO1 tended to decline, indicating suppression of the antioxidant defense pathway under CSD conditions, in line with evidence that sleep disruption can impair Nrf2 signaling [55]. PG elevated the expression of Nrf2 and its downstream targets, indicating improved activation of endogenous antioxidant pathways. This enhancement may contribute to the increased redox capacity observed in PG-treated groups and provides a mechanistic basis for its antioxidative effects. Given that Nrf2 activity can negatively regulate pro-inflammatory gene expression, enhanced Nrf2 signaling may also contribute to the observed reduction in NF-κB activation.

Inflammatory cytokines critically regulate the neuroimmune response during persistent stress [56]. Pro-inflammatory factors such as IL-1β, IL-6, and TNF-α promote microglial activation and disrupt synaptic function, leading to neuronal injury [57]. These cytokines operate within an interconnected signaling network, in which TNF-α and IL-1β activate NF-κB signaling in microglia to induce further cytokine release, thereby forming a self-perpetuating inflammatory cycle that contributes to synaptic impairment [58]. Consistent with previous reports [59], CSD markedly elevated these cytokines in the hippocampus, indicating a persistent pro-inflammatory milieu that contributes to the observed neurobehavioral deficits. PG administration attenuated this inflammatory response, as evidenced by lower cytokine expression in the hippocampus, suggesting that PG alleviates CSD-induced neuroinflammation. Such anti-inflammatory effects of PG align with its impact on NF-κB signaling and support its potential role in disrupting cytokine-driven inflammatory amplification. Comparable reductions in inflammatory cytokines have also been observed with other polyphenol-based interventions in sleep-deprivation models [60].

At the molecular level, the NF-κB signaling pathway serves as a pivotal transcriptional regulator of neuroinflammation [61]. Under stress conditions, excessive ROS generation and cytokine stimulation promote the phosphorylation and degradation of IκBα, the inhibitory subunit that normally sequesters NF-κB in the cytoplasm [62]. After liberation from its inhibitory complex, the NF-κB p65/p50 dimer moves into the nucleus, where it recognizes κB regulatory sequences and activates the transcription of a broad set of pro-inflammatory genes [63]. Among these, iNOS and COX2 act as major effector enzymes responsible for the overproduction of nitric oxide and prostaglandins, thereby amplifying oxidative and inflammatory injury within neural tissue [64]. Consistent with previous reports showing that CSD activates NF-κB and up-regulates its downstream mediators [65], our results demonstrate that PG administration suppressed NF-κB activation and reduced iNOS and COX2 expression. These molecular changes further support the anti-inflammatory actions of PG and indicate that modulation of NF-κB activation is accompanied by altered expression of its downstream pro-inflammatory target genes, including iNOS and COX2.

Oxidative stress and inflammation are tightly interconnected processes that collectively drive neuronal dysfunction during CSD. In this study, CSD induced concurrent oxidative and inflammatory responses, as reflected by decreased antioxidant enzyme activity and elevated cytokine expression accompanied by NF-κB activation. PG treatment enhanced Nrf2-mediated antioxidant defenses and attenuated NF-κB-dependent inflammatory signaling, thereby disrupting the pathological interaction between oxidative damage and neuroinflammation. Importantly, this study provides the first integrated evidence that a lipophilic gallic acid derivative can simultaneously modulate Nrf2 and NF-κB signaling in a CSD model, highlighting a dual-pathway regulatory mechanism underlying neuroprotection. By linking molecular signaling outcomes with behavioral improvements, this work offers a more comprehensive perspective on the neuroprotective potential of PG. Taken together, these findings identify PG as a multifunctional neuroprotective compound that supports neural homeostasis under CSD. Nevertheless, the present study mainly emphasizes integrated behavioral outcomes and related molecular changes, and further work is needed to expand the scope of these observations. Future studies are warranted to examine dose–response patterns in greater detail and to explore the translational potential of PG in sleep-related cognitive disorders.

## 5. Conclusions

This study demonstrates that PG, a lipophilic ester of gallic acid, effectively counteracts the cognitive and molecular disturbances induced by CSD. PG improved multiple domains of cognitive performance—including exploratory behavior, recognition memory, passive-avoidance retention, and spatial learning—while simultaneously restoring redox homeostasis and suppressing neuroinflammatory activation. At the molecular level, PG enhanced Nrf2–HO-1–NQO1 antioxidant signaling and attenuated NF-κB-mediated inflammatory responses, thereby disrupting the pathological interaction between oxidative stress and neuroinflammation. These findings highlight PG as a multifunctional neuroprotective compound capable of preserving hippocampal integrity and cognitive function under conditions of prolonged sleep loss.

## Figures and Tables

**Table 1:** Effects of PG on total swimming distance in MWM in mice subjected to CSD. Data are expressed as mean ± SD (*n* = 10). Total swimming distance is expressed in millimeters (mm). ^#^ *p* < 0.05, ^##^ *p* < 0.01, ^####^ *p* < 0.0001 vs. CON; ** *p* < 0.01, *** *p* < 0.001, **** *p* < 0.0001 vs. CSD.

Group	D1	D2	D3	D4	D5
CON	15,498.95 ± 4.03	14,831.6 ± 38.04	14,307.95 ± 175.29	10,360.55 ± 70.5	8310.7 ± 276.62
CSD	16,204.95 ± 158.89 ^##^	15,429.45 ± 91.99 ^#^	14,885.85 ± 299.32 ^#^	13,282.85 ± 33.16 ^####^	12,266.2 ± 212.27 ^####^
GBE	16,344.65 ± 51.55	15,732.65 ± 81.81	14,345.7 ± 59.11	9756.6 ± 197.14 ****	7206.85 ± 148.28 ****
PG 50 mg/kg	16,721.75 ± 153.23	14,690.05 ± 21 **	13,252.85 ± 62.72 ****	11,522.8 ± 245.79 ****	9437.85 ± 295.22 ****
PG 100 mg/kg	15,733.95 ± 99.21	14,504.5 ± 168.57 ***	13,490.8 ± 291.89 ****	10,659.7 ± 276.05 ****	9373.75 ± 58.62 ****
PG 200 mg/kg	15,306.05 ± 274.29 ***	14,510.65 ± 300.59 ***	12,338.5 ± 259.23 ****	11,193.75 ± 33.45 ****	9889.45 ± 125.79 ****

**Table 2:** Effects of PG on escape latency during the MWM training sessions in mice exposed to CSD. Data are expressed as mean ± SD (*n* = 10). Escape latency is expressed in seconds (s). ^####^ *p* < 0.0001 vs. CON; ** *p* < 0.01, **** *p* < 0.0001 vs. CSD.

Group	D1	D2	D3	D4	D5
CON	60.05 ± 0.07	53.05 ± 0.49	45 ± 0.42	35.65 ± 1.2	22.2 ± 1.7
CSD	60.05 ± 0.07	56.2 ± 5.37	45 ± 2.69	48.8 ± 1.41 ^####^	44.05 ± 1.91 ^####^
GBE	60.05 ± 0.07	57.05 ± 4.17	36.85 ± 4.17 **	34.6 ± 1.13 ****	24.25 ± 0.92 ****
PG 50 mg/kg	60.05 ± 0.07	53.5 ± 1.41	45.5 ± 4.1	32.1 ± 0.71 ****	14.5 ± 0.14 ****
PG 100 mg/kg	60 ± 0	54.6 ± 1.84	44.55 ± 3.04	32 ± 0.71 ****	17.8 ± 0.42 ****
PG 200 mg/kg	60.05 ± 0.07	50.6 ± 0.42	31.5 ± 1.13 ****	21.05 ± 2.47 ****	16.15 ± 3.32 ****

**Table 3:** Effects of PG on quadrant dwell time in the MWM probe trial in CSD-induced cognitive impairment. Data are expressed as mean ± SD (*n* = 10). Dwell time in each quadrant is expressed in seconds (s). ^###^ *p* < 0.001 vs. CON; * *p* < 0.05, ** *p* < 0.01, *** *p* < 0.001, **** *p* < 0.0001 vs. CSD.

Group	Q1	Q2	Q3	Q4
CON	26.85 ± 1.06	16.3 ± 0.85	16.7 ± 1.27	21.15 ± 0.64
CSD	18.15 ± 0.92	25.7 ± 3.96	34.65 ± 5.73 ^###^	30.1 ± 5.23
GBE	37.45 ± 10.25 ****	18.65 ± 2.76	18.55 ± 0.21 ***	23.7 ± 1.41
PG 50 mg/kg	32.3 ± 2.55 **	19.55 ± 0.64	17.9 ± 1.41 ***	18.85 ± 1.91 *
PG 100 mg/kg	28.75 ± 2.47 *	20.35 ± 4.17	21.6 ± 0.42 **	28.65 ± 2.62
PG 200 mg/kg	22.25 ± 0.92	16.9 ± 0.85	18.85 ± 2.76 ***	26.75 ± 3.04

## Data Availability

The original contributions presented in the study are included in the article; further inquiries can be directed to the corresponding authors.

## Supplementary Materials

The following supporting information can be downloaded at: https://www.mdpi.com/article/10.3390/antiox15010079/s1.

## Abbreviations

The following abbreviations are used in this manuscript:

PG	Propyl gallate
CSD	Chronic Sleep Deprivation
GBE	Ginkgo biloba Extract
OFT	Open Field Test
NOR	Novel Object Recognition
ST	Step-Through
MWM	Morris Water Maze
ARE	Antioxidant Response Element
T-AOC	Total Antioxidant Capacity
SOD	Superoxide Dismutase
MDA	Malondialdehyde
TBARS	Thiobarbituric Acid Reactive Substances
Nrf2	Nuclear Factor Erythroid 2–Related Factor 2
HO-1	Heme Oxygenase-1
NQO1	NAD(P)H Quinone Oxidoreductase 1
NF-κB	Nuclear Factor Kappa-B
iNOS	Inducible Nitric Oxide Synthase
COX2	Cyclooxygenase-2
IL-1β	Interleukin-1β
IL-6	Interleukin-6
TNF-α	Tumor Necrosis Factor-α
