# Supplementary material for: Propyl Gallate Attenuates Cognitive Deficits Induced by Chronic Sleep Deprivation Through Nrf2 Activation and NF-κB Inhibition
Source: Antioxidants (Basel). 2026 Jan 7;15(1):79. doi: 10.3390/antiox15010079 (PMC12837502; doi:10.3390/antiox15010079)
Supplement: Supplementary file 1 [file antioxidants-15-00079-s001.zip › antioxidants-4040106-supplementary.pdf]

**Table S1. Average speed in the open field test: mean, SD, and sample size (n = 10 per group).**

| Group | Mean | SD | N |
|---|---|---|---|
| **CON** | 57.10 | 10.74 | 10 |
| **CSD** | 41.19 | 8.83 | 10 |
| **GBE** | 59.22 | 14.26 | 10 |
| **PG-50mg/kg** | 54.40 | 13.49 | 10 |
| **PG-100mg/kg** | 65.09 | 16.24 | 10 |
| **PG-200mg/kg** | 59.61 | 18.62 | 10 |

**Table S2. Total movement distance in the open field test: mean, SD, and sample size (n = 10 per group).**

| Group | Mean | SD | N |
|---|---|---|---|
| **CON** | 350.97 | 61.91 | 10 |
| **CSD** | 244.12 | 56.99 | 10 |
| **GBE** | 352.29 | 90.27 | 10 |
| **PG-50mg/kg** | 344.50 | 122.55 | 10 |
| **PG-100mg/kg** | 380.53 | 46.13 | 10 |
| **PG-200mg/kg** | 360.18 | 127.50 | 10 |

**Table S3. Center-to-periphery time ratio in the open field test: mean, SD, and sample size (n = 10 per group).**

| Group | Mean | SD | N |
|---|---|---|---|
| **CON** | 0.24 | 0.07 | 10 |
| **CSD** | 0.19 | 0.07 | 10 |
| **GBE** | 0.27 | 0.08 | 10 |
| **PG-50mg/kg** | 0.25 | 0.06 | 10 |
| **PG-100mg/kg** | 0.19 | 0.07 | 10 |
| **PG-200mg/kg** | 0.20 | 0.10 | 10 |

**Table S4. Total exploration time in the novel object recognition test: mean, SD, and sample size (n = 10 per group).**

| Group | Mean | SD | N |
|---|---|---|---|
| **CON** | 260.52 | 35.40 | 10 |
| **CSD** | 187.08 | 60.62 | 10 |
| **GBE** | 254.12 | 79.68 | 10 |
| **PG-50mg/kg** | 216.92 | 67.06 | 10 |
| **PG-100mg/kg** | 283.96 | 90.57 | 10 |
| **PG-200mg/kg** | 228.03 | 98.49 | 10 |

**Table S5. Exploration time of familiar (A1) and novel (B) objects in the novel object recognition test: mean, SD, and sample size (n = 10 per group).**

| Group(A1) | Mean | SD | N |
|---|---|---|---|
| CON | 86.86 | 55.59 | 10 |
| CSD | 106.91 | 59.05 | 10 |
| GBE | 135.91 | 97.59 | 10 |
| PG-50mg/kg | 103.86 | 85.03 | 10 |
| PG-100mg/kg | 115.09 | 60.43 | 10 |
| PG-200mg/kg | 69.71 | 44.65 | 10 |

| Group(B) | Mean | SD | N |
|---|---|---|---|
| CON | 103.66 | 47.76 | 10 |
| CSD | 103.37 | 83.56 | 10 |
| GBE | 113.21 | 72.91 | 10 |
| PG-50mg/kg | 103.66 | 72.20 | 10 |
| PG-100mg/kg | 128.87 | 76.37 | 10 |
| PG-200mg/kg | 98.32 | 74.91 | 10 |

**Table S6. Discrimination index in the novel object recognition test: mean, SD, and sample size (n = 10 per group).**

| Group | Mean | SD | N |
|---|---|---|---|
| CON | 0.53 | 0.13 | 10 |
| CSD | -0.17 | 0.17 | 10 |
| GBE | 0.41 | 0.12 | 10 |
| PG-50mg/kg | 0.32 | 0.15 | 10 |
| PG-100mg/kg | 0.37 | 0.20 | 10 |
| PG-200mg/kg | 0.26 | 0.09 | 10 |

**Table S7. Number of errors in the behavioral task: mean, SD, and sample size (n = 10 per group).**

| Group | Mean | SD | N |
|---|---|---|---|
| CON | 3.00 | 1.25 | 10 |
| CSD | 5.80 | 1.75 | 10 |
| GBE | 3.20 | 1.62 | 10 |
| PG-50mg/kg | 3.90 | 1.37 | 10 |
| PG-100mg/kg | 4.00 | 2.00 | 10 |
| PG-200mg/kg | 3.80 | 1.69 | 10 |

**Table S8. Latency period in the behavioral task: mean, SD, and sample size (n = 10 per group).**

| Group | Mean | SD | N |
|---|---|---|---|
| **CON** | 67.22 | 15.94 | 10 |
| **CSD** | 26.06 | 18.09 | 10 |
| **GBE** | 77.34 | 14.46 | 10 |
| **PG-50mg/kg** | 57.92 | 36.21 | 10 |
| **PG-100mg/kg** | 74.67 | 22.16 | 10 |
| **PG-200mg/kg** | 76.22 | 16.42 | 10 |

**Table S9. Swimming speed in the behavioral test: mean, SD, and sample size (n = 10 per group).**

| Group | Mean | SD | N |
|---|---|---|---|
| **CON** | 26.31 | 3.57 | 10 |
| **CSD** | 13.69 | 2.23 | 10 |
| **GBE** | 32.69 | 10.18 | 10 |
| **PG-50mg/kg** | 20.71 | 3.95 | 10 |
| **PG-100mg/kg** | 21.62 | 8.00 | 10 |
| **PG-200mg/kg** | 23.21 | 3.36 | 10 |

**Table S10. Crossing number in the behavioral test: mean, SD, and sample size (n = 10 per group).**

| Group | Mean | SD | N |
|---|---|---|---|
| **CON** | 3.30 | 1.16 | 10 |
| **CSD** | 0.80 | 0.79 | 10 |
| **GBE** | 3.20 | 1.14 | 10 |
| **PG-50mg/kg** | 1.90 | 0.99 | 10 |
| **PG-100mg/kg** | 2.00 | 0.67 | 10 |
| **PG-200mg/kg** | 2.80 | 1.03 | 10 |

**Table S11. Serum total antioxidant capacity: mean, SD, and sample size (n = 6 per group).**

| Group | Mean | SD | N |
|---|---|---|---|
| **CON** | 0.61 | 0.09 | 6 |
| **CSD** | 0.42 | 0.07 | 6 |
| **GBE** | 0.62 | 0.07 | 6 |
| **PG-50mg/kg** | 0.53 | 0.05 | 6 |
| **PG-100mg/kg** | 0.57 | 0.04 | 6 |
| **PG-200mg/kg** | 0.55 | 0.07 | 6 |

**Table S12. Serum superoxide dismutase activity: mean, SD, and sample size (n = 6 per group).**

| Group | Mean | SD | N |
|---|---|---|---|
| **CON** | 22.29 | 4.63 | 6 |
| **CSD** | 14.10 | 2.83 | 6 |
| **GBE** | 20.81 | 3.48 | 6 |
| **PG-50mg/kg** | 20.20 | 3.01 | 6 |
| **PG-100mg/kg** | 20.24 | 4.42 | 6 |
| **PG-200mg/kg** | 20.31 | 3.44 | 6 |

**Table S13. Serum TBARS levels (malondialdehyde equivalents): mean, SD, and sample size (n = 6 per group).**

| Group | Mean | SD | N |
|---|---|---|---|
| **CON** | 1.87 | 0.44 | 6 |
| **CSD** | 7.85 | 1.20 | 6 |
| **GBE** | 2.15 | 0.63 | 6 |
| **PG-50mg/kg** | 6.01 | 0.82 | 6 |
| **PG-100mg/kg** | 6.07 | 1.23 | 6 |
| **PG-200mg/kg** | 5.60 | 0.83 | 6 |

**Table S14. Hippocampal total antioxidant capacity: mean, SD, and sample size (n = 6 per group).**

| Group | Mean | SD | N |
|---|---|---|---|
| **CON** | 4.14 | 0.48 | 6 |
| **CSD** | 2.83 | 0.64 | 6 |
| **GBE** | 3.84 | 0.39 | 6 |
| **PG-50mg/kg** | 3.58 | 0.38 | 6 |
| **PG-100mg/kg** | 3.60 | 0.35 | 6 |
| **PG-200mg/kg** | 3.54 | 0.46 | 6 |

**Table S15. Hippocampal superoxide dismutase activity: mean, SD, and sample size (n = 6 per group).**

| Group | Mean | SD | N |
|---|---|---|---|
| **CON** | 152.98 | 13.66 | 6 |
| **CSD** | 115.32 | 13.14 | 6 |
| **GBE** | 160.07 | 13.63 | 6 |
| **PG-50mg/kg** | 124.71 | 11.24 | 6 |
| **PG-100mg/kg** | 130.52 | 11.72 | 6 |
| **PG-200mg/kg** | 140.13 | 7.16 | 6 |

**Table S16. Hippocampal TBARS levels (malondialdehyde equivalents): mean, SD, and sample size (n = 6 per group).**

| Group | Mean | SD | N |
|---|---|---|---|
| CON | 8.72 | 1.48 | 6 |
| CSD | 17.69 | 2.79 | 6 |
| GBE | 11.24 | 2.10 | 6 |
| PG-50mg/kg | 13.75 | 1.81 | 6 |
| PG-100mg/kg | 14.19 | 2.13 | 6 |
| PG-200mg/kg | 14.38 | 1.68 | 6 |

**Table S17. Relative protein expression of Nrf2 normalized to β-actin: mean, SD, and sample size (n = 3 per group).**

| Group | Mean | SD | N |
|---|---|---|---|
| CON | 1.13 | 0.09 | 3 |
| CSD | 0.27 | 0.11 | 3 |
| GBE | 1.06 | 0.10 | 3 |
| PG-50mg/kg | 0.19 | 0.05 | 3 |
| PG-100mg/kg | 0.38 | 0.04 | 3 |
| PG-200mg/kg | 0.55 | 0.04 | 3 |

**Table S18. Relative protein expression of HO-1 normalized to β-actin: mean, SD, and sample size (n = 3 per group).**

| Group | Mean | SD | N |
|---|---|---|---|
| CON | 1.16 | 0.04 | 3 |
| CSD | 0.20 | 0.06 | 3 |
| GBE | 1.05 | 0.15 | 3 |
| PG-50mg/kg | 0.61 | 0.09 | 3 |
| PG-100mg/kg | 0.86 | 0.09 | 3 |
| PG-200mg/kg | 0.37 | 0.06 | 3 |

**Table S19. Relative protein expression of NQO1 normalized to β-actin: mean, SD, and sample size (n = 3 per group).**

| Group | Mean | SD | N |
|---|---|---|---|
| CON | 1.09 | 0.07 | 3 |
| CSD | 0.24 | 0.08 | 3 |
| GBE | 1.08 | 0.06 | 3 |
| PG-50mg/kg | 0.35 | 0.06 | 3 |
| PG-100mg/kg | 0.79 | 0.06 | 3 |
| PG-200mg/kg | 0.86 | 0.03 | 3 |

**Table S20. Serum interleukin-1β content: mean, SD, and sample size (n = 6 per group).**

| Group | Mean | SD | N |
|---|---|---|---|
| **CON** | 11.94 | 2.05 | 6 |
| **CSD** | 24.66 | 2.53 | 6 |
| **GBE** | 14.62 | 2.32 | 6 |
| **PG-50mg/kg** | 17.25 | 2.03 | 6 |
| **PG-100mg/kg** | 18.72 | 3.49 | 6 |
| **PG-200mg/kg** | 19.05 | 2.74 | 6 |

**Table S21. Serum interleukin-6 content: mean, SD, and sample size (n = 6 per group).**

| Group | Mean | SD | N |
|---|---|---|---|
| **CON** | 2.77 | 0.72 | 6 |
| **CSD** | 7.44 | 1.63 | 6 |
| **GBE** | 2.71 | 0.69 | 6 |
| **PG-50mg/kg** | 5.52 | 1.02 | 6 |
| **PG-100mg/kg** | 3.51 | 0.71 | 6 |
| **PG-200mg/kg** | 5.07 | 1.10 | 6 |

**Table S22. Serum tumor necrosis factor-α content: mean, SD, and sample size (n = 6 per group).**

| Group | Mean | SD | N |
|---|---|---|---|
| **CON** | 85.26 | 9.88 | 6 |
| **CSD** | 142.79 | 16.49 | 6 |
| **GBE** | 76.97 | 14.26 | 6 |
| **PG-50mg/kg** | 107.06 | 13.65 | 6 |
| **PG-100mg/kg** | 114.30 | 10.47 | 6 |
| **PG-200mg/kg** | 107.35 | 14.44 | 6 |

**Table S23. Hippocampal interleukin-1β content: mean, SD, and sample size (n = 6 per group).**

| Group | Mean | SD | N |
|---|---|---|---|
| **CON** | 17.73 | 2.79 | 6 |
| **CSD** | 29.56 | 3.73 | 6 |
| **GBE** | 14.10 | 2.32 | 6 |
| **PG-50mg/kg** | 20.91 | 3.93 | 6 |
| **PG-100mg/kg** | 22.17 | 4.07 | 6 |
| **PG-200mg/kg** | 20.41 | 3.72 | 6 |

**Table S24. Hippocampal interleukin-6 content: mean, SD, and sample size (n = 6 per group).**

| Group | Mean | SD | N |
|---|---|---|---|
| CON | 16.55 | 5.34 | 6 |
| CSD | 32.63 | 6.42 | 6 |
| GBE | 17.65 | 4.50 | 6 |
| PG-50mg/kg | 24.68 | 3.06 | 6 |
| PG-100mg/kg | 24.87 | 4.64 | 6 |
| PG-200mg/kg | 24.98 | 3.97 | 6 |

**Table S25. Hippocampal tumor necrosis factor-α content: mean, SD, and sample size (n = 6 per group).**

| Group | Mean | SD | N |
|---|---|---|---|
| CON | 73.28 | 15.39 | 6 |
| CSD | 131.67 | 15.85 | 6 |
| GBE | 92.59 | 13.05 | 6 |
| PG-50mg/kg | 107.12 | 14.43 | 6 |
| PG-100mg/kg | 106.82 | 12.81 | 6 |
| PG-200mg/kg | 107.58 | 18.90 | 6 |

**Table S26. Relative protein expression of p-p65 normalized to p65: mean, SD, and sample size (n = 3 per group).**

| Group | Mean | SD | N |
|---|---|---|---|
| CON | 0.29 | 0.02 | 3 |
| CSD | 1.22 | 0.10 | 3 |
| GBE | 0.37 | 0.05 | 3 |
| PG-50mg/kg | 0.93 | 0.06 | 3 |
| PG-100mg/kg | 0.68 | 0.07 | 3 |
| PG-200mg/kg | 0.52 | 0.04 | 3 |

**Table S27. Relative protein expression of iNOS normalized to β-actin: mean, SD, and sample size (n = 3 per group).**

| Group | Mean | SD | N |
|---|---|---|---|
| CON | 0.17 | 0.03 | 3 |
| CSD | 1.02 | 0.12 | 3 |
| GBE | 0.18 | 0.07 | 3 |
| PG-50mg/kg | 0.36 | 0.10 | 3 |
| PG-100mg/kg | 0.36 | 0.08 | 3 |
| PG-200mg/kg | 0.32 | 0.08 | 3 |

**Table S28. Relative protein expression of COX2 normalized to β-actin: mean, SD, and sample size (n = 3 per group).**

| Group | Mean | SD | N |
|---|---|---|---|
| **CON** | 0.18 | 0.03 | 3 |
| **CSD** | 0.96 | 0.16 | 3 |
| **GBE** | 0.26 | 0.09 | 3 |
| **PG-50mg/kg** | 0.29 | 0.10 | 3 |
| **PG-100mg/kg** | 0.49 | 0.14 | 3 |
| **PG-200mg/kg** | 0.51 | 0.13 | 3 |
